# Association of N-terminal Pro-B-Type Natriuretic Peptide Levels and Electrocardiogram Changes With the Severity of Airflow Narrowing and Exercise Tolerance in Stable Chronic Obstructive Pulmonary Disease Patients: A Cross-Sectional Analytical Study

**DOI:** 10.7759/cureus.99084

**Published:** 2025-12-12

**Authors:** Thannushree Aritakulu Badrinath, Arun Prasath, Pinkutty Sagar, Antonious Selvam

**Affiliations:** 1 Respiratory Medicine, Pondicherry Institute of Medical Sciences, Puducherry, IND; 2 Pulmonology, Pondicherry Institute of Medical Sciences, Puducherry, IND; 3 Pulmonary Medicine, Pondicherry Institute of Medical Sciences, Puducherry, IND

**Keywords:** 6mwt, cardiopulmonary dysfunction, chronic obstructive pulmonary disease (copd), ecg (electrocardiogram), electrocardiogram (ecg), nt-probnp

## Abstract

Background: Chronic obstructive pulmonary disease (COPD) is associated with significant cardiovascular morbidity. N-terminal pro-B-type natriuretic peptide (NT-proBNP), electrocardiogram (ECG) abnormalities, and reduced exercise tolerance may reflect cardiac involvement in COPD, but their relationship with airflow limitation stages is incompletely defined. This study evaluated associations between NT-proBNP, ECG changes, and six-minute walk distance (6MWD) across the Global Initiative for Obstructive Lung Disease (GOLD) stages in stable COPD patients.

Methods: A hospital-based cross-sectional analytical study enrolled 88 stable COPD patients aged ≥40 years between October 2022 and June 2024. Demographic, clinical, spirometric, ECG, 6MWD, and NT-proBNP data were collected. COPD severity was classified by the GOLD criteria. Continuous variables were compared by analysis of variance (ANOVA) and categorical variables by the chi-squared test. A p-value of <0.05 indicated statistical significance.

Results: Of 88 participants, 71 (80.7%) were male and 17 (19.3%) female. Distribution by GOLD stage was as follows: GOLD I 20 (22.7%), GOLD II 39 (44.3%), GOLD III 22 (25%), and GOLD IV seven (8%). Exercise capacity declined with severity: the mean 6MWD decreased markedly from the mild to the very severe groups. NT-proBNP levels rose progressively with airflow limitation (ANOVA p < 0.001), with markedly higher mean concentrations in the very severe group. ECG abnormalities increased with disease stage: P pulmonale was present in 24 (27.3%), right ventricular hypertrophy in 27 (30.7%), right axis deviation in 27 (30.7%), low voltage complexes in eight (9.1%), and incomplete right bundle branch block (IRBBB) in five (5.7%) of the cohort; these changes were more frequent in severe/very severe COPD. Smoking exposure and indices (current/former smokers, cigarettes/day, smoking index, duration) showed significant upward trends with worsening GOLD stage (p ≤ 0.042). NT-proBNP correlated positively with COPD stage, while 6MWD correlated negatively (r values significant; p < 0.001).

Conclusion: In stable COPD, advancing airflow limitation is associated with rising NT-proBNP, increasing ECG evidence of right heart strain, and declining exercise tolerance. NT-proBNP and simple ECG markers, alongside functional testing, may aid the early detection of cardiovascular involvement in COPD and help risk-stratify patients for closer cardiopulmonary evaluation and management.

## Introduction

Chronic obstructive pulmonary disease (COPD) is a major global health concern characterized by persistent, progressive airflow limitation and an abnormal inflammatory response of the lungs to harmful particles or gases [1]. It primarily includes chronic bronchitis and emphysema, which together cause irreversible damage to the airways and lung parenchyma, leading to respiratory insufficiency and impaired quality of life [2]. Globally, an estimated 65 million people suffer from moderate to severe COPD, with approximately 4.5 million deaths occurring annually due to its complications [3]. Currently ranked as the third leading cause of death worldwide, COPD not only poses a significant health burden but also contributes substantially to socioeconomic costs through repeated hospitalizations, medication expenses, and productivity loss [4].

Parallel to this, cardiovascular disease (CVD) remains a silent yet pervasive threat to public health. It encompasses a range of disorders involving the heart and blood vessels, including coronary artery disease, heart failure, arrhythmias, and stroke, which together account for a large share of global mortality [5]. Despite advances in medical care, CVD continues to be a major cause of premature death worldwide. Its multifactorial etiology involves genetic predisposition, smoking, hypertension, diabetes, sedentary lifestyle, and environmental stressors [6]. The complex pathophysiological mechanisms underlying CVD highlight the need for early detection and integrated management strategies to reduce its global burden.

COPD and CVD frequently coexist, posing intersecting challenges in global health. Both share common risk factors such as age, smoking, systemic inflammation, and oxidative stress, making their coexistence clinically significant [7]. Studies indicate that cardiac complications are the most frequent cause of death among COPD patients, rather than respiratory failure [7,8]. The chronic hypoxia and systemic inflammation in COPD can aggravate endothelial dysfunction, pulmonary hypertension, and atherosclerosis, predisposing patients to cardiac arrhythmias and heart failure [9]. Hence, identifying cardiovascular involvement early in the course of COPD could significantly improve prognosis and management outcomes.

Natriuretic peptides are cardiac neurohormones that play a crucial role in regulating sodium balance, vascular tone, and fluid homeostasis [10]. They are primarily secreted in response to ventricular stretch or pressure overload. There are three main types: atrial natriuretic peptide (ANP), brain natriuretic peptide (BNP), and C-type natriuretic peptide (CNP). Among them, B-type natriuretic peptide (BNP) and its inactive fragment N-terminal pro-B-type natriuretic peptide (NT-proBNP) are well-established biomarkers for ventricular dysfunction and cardiac stress. Elevated NT-proBNP levels have been reported in patients with left ventricular hypertrophy, pulmonary hypertension, and right ventricular overload, conditions often associated with chronic lung disease [11]. Thus, measurement of plasma NT-proBNP offers a reliable, non-invasive indicator for detecting subclinical cardiac dysfunction in COPD patients. However, the relationship between NT-proBNP levels and the severity of airflow obstruction in stable COPD remains insufficiently explored [9-11].

The six-minute walk test (6MWT) is a simple, inexpensive, and reproducible method to assess exercise tolerance and functional capacity in COPD patients [12]. It evaluates the distance a patient can walk in six minutes on a flat surface, providing an integrative measure of cardiopulmonary and muscular function. Unlike forced expiratory volume in one second (FEV₁), which quantifies airflow obstruction, the 6MWT reflects the patient's overall functional status and ability to perform daily activities. Combining spirometric parameters with functional assessments such as the 6MWT provides a more comprehensive understanding of disease severity, physical limitations, and response to therapy.

The electrocardiogram (ECG) is a rapid, inexpensive, and non-invasive diagnostic tool for evaluating cardiac abnormalities in COPD. Typical ECG changes seen in COPD include right axis deviation (RAD), P pulmonale, poor R-wave progression, low voltage complexes, right bundle branch block (RBBB), and right ventricular hypertrophy (RVH) [13]. These findings reflect chronic hypoxemia, pulmonary hypertension, and right ventricular strain secondary to long-standing airflow limitation. Among these, P pulmonale, RAD, and RVH are the most consistent and correlate with increasing disease severity. Despite its diagnostic value, ECG evaluation is often underutilized in COPD care, and the relationship between NT-proBNP levels, ECG changes, and airflow obstruction severity has not been comprehensively studied.

The coexistence of COPD and CVD represents a synergistic burden that amplifies morbidity and mortality. Understanding their interrelationship is critical for improving diagnostic accuracy, guiding management, and preventing adverse outcomes. As the majority of COPD-related deaths stem from cardiac complications, early identification of cardiovascular involvement can play a transformative role in patient care. Therefore, the primary objective of the present study is to evaluate the association between NT-proBNP levels, ECG changes, and exercise tolerance with the stages of airflow narrowing in stable COPD patients. 

## Materials and methods

Study setting

The present hospital-based cross-sectional analytical study was conducted at the Pondicherry Institute of Medical Sciences (PIMS), a 740-bedded multi-specialty teaching hospital in Pondicherry. The institute caters to patients from both Pondicherry and neighboring districts of Tamil Nadu, primarily serving rural farming and fishing communities. The study was carried out in the Department of Respiratory Medicine between October 2022 and June 2024. The objective was to assess stable COPD patients diagnosed as per the Global Initiative for Obstructive Lung Disease (GOLD) 2022 guidelines, with a focus on their ECG and biochemical profiles. Institutional ethical committee approval was obtained prior to the initiation of the study.

Study design and period

This was a hospital-based cross-sectional analytical study conducted over a period of 20 months, from October 2022 to June 2024. The study was approved by the PIMS Institutional Ethics Committee (approval number: IEC: RC/2022/100). The design facilitated the assessment of COPD patients in a stable phase through clinical, spirometric, radiological, and biochemical evaluations to determine associations between cardiopulmonary parameters and disease severity.

Study participants

The study population comprised stable COPD patients attending the Respiratory Medicine Outpatient Department (OPD) at PIMS. Patients were recruited after meeting predefined inclusion and exclusion criteria.

Inclusion and Exclusion Criteria

Stable COPD patients aged 40 years or above diagnosed according to the GOLD 2022 criteria were included. Patients with acute exacerbation of COPD or acute coronary syndrome within the past four weeks, with other chronic respiratory diseases, or with significant comorbidities involving cardiac, renal, hepatic, neurological, or psychological systems were excluded. Additionally, patients with marked hemodynamic instability, uncontrolled diabetes mellitus and hypertension, malignancy, or pregnancy were not included.

Sample size and sampling technique

The sample size was calculated based on the study "Prognostic value of plasma brain natriuretic peptide in patients with stable chronic obstructive pulmonary disease" by Mansour et al. [14]. Using a correlation coefficient (r) of -0.489, a power of 95%, an alpha error of 5%, and a two-sided test, the minimum sample size was calculated as 88 stable COPD patients. The study employed a consecutive sampling technique, and all eligible patients attending the OPD during the study period who provided informed consent were enrolled.

Study tools and data collection

Data were collected using a structured Case Report Form (CRF) designed for the study. Instruments used included a stadiometer, weighing scale, pulse oximeter, thermometer, blood pressure apparatus, and stethoscope. Diagnostic and laboratory tools comprised spirometry, chest X-ray (posteroanterior view), 12-lead ECG, and blood investigations including urea, creatinine, and NT-proBNP estimation. Each participant underwent a detailed history, general and systemic examination, spirometry, diffusion capacity assessment (diffusing capacity of the lungs for carbon monoxide (DLCO)), and 6MWT following standard protocols.

Study variables

Sociodemographic Variables

Age, gender, and occupation were recorded for each participant.

Clinical Variables

Variables included grades of dyspnea (modified Medical Research Council scale), past exacerbations, comorbidities, tobacco exposure (pack-years), biomass fuel exposure (biomass index), body mass index (BMI), pulse rate, respiratory rate, blood pressure, and oxygen saturation.

Investigative Variables

Radiological findings (chest X-ray), ECG parameters (rhythm, axis, P-wave, QRS complex, PR and RR intervals, QTc), spirometric indices (FEV₁, forced vital capacity (FVC), FEV₁/FVC ratio, forced expiratory flow (FEF)25-75%, DLCO, total lung capacity (TLC)), peak expiratory flow rate (PEFR), 6MWT distance, and NT-proBNP levels were also documented.

Operational definitions

COPD was defined as per the GOLD 2022 guidelines as a common, preventable, and treatable disease characterized by persistent respiratory symptoms and airflow limitation due to exposure to noxious particles or gases. Diagnosis required post-bronchodilator spirometry showing an FEV₁/FVC ratio <0.70 in symptomatic patients aged over 40 years with significant exposure to risk factors such as tobacco smoke or biomass fuel. Dyspnea was described as progressive, persistent, and typically worse on exertion. Other defining features included chronic cough, sputum production, and recurrent respiratory infections.

ECG diagnostic criteria

The ECG abnormalities were defined using standard diagnostic parameters. P pulmonale was identified when the P-wave amplitude exceeded 2.5 mm in inferior leads II, III, and augmented vector foot (aVF). RBBB was diagnosed if QRS duration exceeded 120 ms with an RSR' pattern in leads V1-V3 and a wide slurred S wave in lateral leads (I, augmented vector left (aVL), V5, V6). Low voltage complexes were identified when the amplitude of the QRS complex was less than 5 mm in three consecutive leads. RAD was defined as a QRS axis >90°, with dominant R waves in II, III, and aVF and a dominant S wave in I. RVH was diagnosed by the presence of RAD along with features such as P pulmonale, R<S in V6, and A+R-P≥0.7, where A is the maximum RR' amplitude in V1/V2, R is the maximal S in I/V6, and P is the minimal S in V1 or minimal R in V6.

Method of performing spirometry

Spirometry was performed in accordance with standardized guidelines. The spirometer was calibrated before each session. Patients were seated upright with a nose clip to prevent nasal air leakage. After clear instructions, patients performed a maximal inspiration followed by a forceful and sustained expiration through the mouthpiece. Three acceptable maneuvers were recorded, and the best reading was considered for analysis. Parameters measured included FEV₁, FVC, FEV₁/FVC, and FEF25-75%. Care was taken to avoid artifacts and ensure accurate recordings. The results were interpreted using reference values standardized for age, sex, height, and ethnicity.

Method of performing the 6MWT

The 6MWT was conducted following the American Thoracic Society (ATS) guidelines in an indoor corridor at least 30 meters in length, with cones marking the turnaround points. After explaining the procedure, baseline vital signs, heart rate, blood pressure, respiratory rate, and oxygen saturation, were recorded. The patient was instructed to walk as far as possible within six minutes at their own pace. Continuous supervision was maintained, and standardized verbal encouragement was provided. The total distance covered was recorded as a measure of exercise tolerance. Post-test vital signs and perceived exertion (Borg scale) were documented, and patients were allowed adequate rest before concluding the procedure.

Method of performing the NT-proBNP assay

Under aseptic precautions, 3 mL of venous blood was drawn from each participant. Serum was separated, labeled, and stored at -6°C until analysis. NT-proBNP levels were measured using a sandwich enzyme-linked immunosorbent assay (ELISA) technique with double antibodies for greater specificity and sensitivity (LifeSpan Biosciences Inc., LSF23701, Newark, CA). The assay had a detection range of 0.63-40 ng/mL and a sensitivity of 0.38 ng/mL. The principle involved antigen-antibody binding in pre-coated microwells, followed by the sequential addition of biotin-conjugated antibodies, horseradish peroxidase (HRP)-linked avidin, and 3,3',5,5'-tetramethylbenzidine (TMB) substrate. The enzymatic color reaction was terminated with sulfuric acid, and absorbance was read at 450 nm using a microplate reader. Concentrations were extrapolated from the standard curve generated from known standards.

Data collection procedure

After obtaining informed consent, eligible patients underwent detailed history taking, general examination, systemic assessment, and investigations as per protocol. All findings were recorded in the CRF. Spirometry and 6MWT were performed as per standard procedures. Chest radiographs and ECGs were interpreted by experienced clinicians, and serum NT-proBNP analysis was carried out in the institutional laboratory. Each parameter was documented systematically for statistical evaluation.

Statistical analysis

Data were entered into Microsoft Excel (Microsoft Corp., Redmond, WA, USA) and analyzed using IBM SPSS Statistics for Windows, V. 26.0 (IBM Corp., Armonk, NY, USA). Quantitative variables were expressed as mean and standard deviation, whereas qualitative variables were presented as frequency and percentage. Associations between clinical, spirometric, and biochemical parameters with disease severity were analyzed using the chi-squared test for categorical variables and analysis of variance (ANOVA) for continuous variables. A p-value of <0.05 was considered statistically significant. The final analysis aimed to determine the relationship between COPD severity, ECG abnormalities, and NT-proBNP levels, thereby elucidating cardiopulmonary interactions in stable COPD patients.

## Results

Among the 88 participants, males constituted the majority (71, 80.7%), while females accounted for 17 (19.3%). Most participants were aged 60-69 years (37, 42%), followed by those aged ≥70 years (26, 29.6%), 50-59 years (13, 14.8%), 40-49 years (11, 12.5%), and <40 years (one, 1.1%). Regarding occupation, the largest group comprised farmers (35, 39.8%), followed by construction workers (23, 26.1%), daily wage laborers (18, 20.5%), and others (12, 13.6%), which included homemakers (five, 5.7%), small-scale business owners (four, 4.5%), and skilled trades such as electricians and drivers (three, 3.4%) (Table 1).

**Table 1:** Distribution of the study participants by age category, gender, and occupation (n = 88) *Others include homemakers (n = 5), small-scale business owners (n = 4), and skilled trades such as electricians and drivers (n = 3).

Variable	Category	n	%
Gender	Male	71	80.7
	Female	17	19.3
Age category (years)	<40	1	1.1
	40-49	11	12.5
	50-59	13	14.8
	60-69	37	42
	≥70	26	29.6
Occupation	Farmer	35	39.8
	Construction worker	23	26.1
	Daily wage laborer	18	20.5
	Others*	12	13.6

Based on the GOLD classification of airflow obstruction, patients were categorized into four groups according to disease severity. Among them, 20 (22.7%) had mild obstruction (GOLD I) with a mean FEV₁ of 85.45 ± 5.2, 39 (44.3%) had moderate obstruction (GOLD II) with a mean FEV₁ of 67.10 ± 6.6, 22 (25%) had severe obstruction (GOLD III) with a mean FEV₁ of 40.45 ± 7.1, and seven (8%) had very severe obstruction (GOLD IV) with a mean FEV₁ of 25.43 ± 4.9. The majority of patients, 61 (69.3%), thus presented with moderate to severe airflow limitation, indicating a higher burden of advanced disease among the study population (Table 2).

**Table 2:** Distribution of patients according to severity of airflow obstruction (n = 88) GOLD: Global Initiative for Obstructive Lung Disease; FEV₁: forced expiratory volume in one second

Severity (GOLD classification)	Category	Number of patients (n)	Percentage (%)	Mean ± SD, FEV₁ (%)
GOLD I	Mild	20	22.7	85.45 ± 5.2
GOLD II	Moderate	39	44.3	67.10 ± 6.6
GOLD III	Severe	22	25	40.45 ± 7.1
GOLD IV	Very severe	7	8	25.43 ± 4.9

Table 3 summarizes the clinical profile and exercise tolerance parameters across different stages of airflow obstruction among patients with COPD. The COPD Assessment Test (CAT) score showed a significant increasing trend with disease severity, rising from a mean of 9.05 ± 3.42 in the mild group to 26.71 ± 5.95 in the very severe group (F = 42.6; p < 0.001), reflecting a progressive decline in health status. Similarly, the mean six-minute walk distance (6MWD) demonstrated a marked reduction with worsening obstruction, decreasing from 487.22 ± 45.30 m in mild cases to 267.86 ± 58.12 m in very severe cases (F = 39.8; p < 0.001). Oxygen saturation also declined significantly across severity stages (F = 26.9; p < 0.001), showing a negative correlation with disease progression. The mean pulse rate exhibited a modest upward trend, increasing from 81 ± 5.62 bpm in mild cases to 90 ± 8.10 bpm in very severe cases (F = 4.95; p = 0.003). In contrast, BMI and blood pressure (systolic/diastolic blood pressure (SBP/DBP)) did not differ significantly among the groups (p > 0.05). Overall, the findings indicate that symptom burden and exercise limitation worsen proportionally with the severity of airflow obstruction, whereas nutritional and hemodynamic parameters remain relatively stable.

**Table 3:** Clinical profile and exercise tolerance across stages of airflow obstruction ANOVA test *A p-value of <0.05 is statistically significant BMI: body mass index; SBP: systolic blood pressure; DBP: diastolic blood pressure; ANOVA: analysis of variance; CAT: chronic obstructive pulmonary disease (COPD) Assessment Test; SpO₂: oxygen saturation

Variable	Mild (mean ± SD)	Moderate (mean ± SD)	Severe (mean ± SD)	Very severe (mean ± SD)	F-value	P-value
CAT score	9.05 ± 3.42	17.54 ± 5.63	23.73 ± 6.12	26.71 ± 5.95	42.6	<0.001*
Distance (m)	487.22 ± 45.30	448.97 ± 51.26	347.36 ± 62.84	267.86 ± 58.12	39.8	<0.001*
BMI (kg/m²)	22.07 ± 2.14	21.99 ± 2.45	21.09 ± 2.30	20.83 ± 2.52	1.42	0.24
SpO₂ (%)	98 ± 1.15	98 ± 1.25	96 ± 1.63	94 ± 2.02	26.9	<0.001*
Pulse rate (bpm)	81 ± 5.62	83 ± 6.14	86 ± 7.12	90 ± 8.10	4.95	0.003*
SBP (mmHg)	124 ± 12	126± 14	130 ± 13	132 ± 15	1.87	0.14
DBP (mmHg)	66 ± 8	68 ± 9	70 ± 10	72 ± 11	1.72	0.12

Among the 88 patients with COPD, smoking-related parameters demonstrated a clear association with increasing disease severity. The proportion of current or former smokers progressively increased from 13 (65%) in mild, 30 (76.9%) in moderate, and 19 (86.4%) in severe to seven (100%) in very severe COPD (χ² = 6.84; p = 0.042). The mean number of cigarettes smoked per day rose from 3.8 ± 1.4 in mild to 6.9 ± 2.1, 8.4 ± 2.6, and 9.1 ± 2.8 in moderate, severe, and very severe groups, respectively, showing a significant upward trend (F = 14.62; p < 0.001). Similarly, the mean smoking index increased markedly from 66.75 ± 25.8 in mild to 213.33 ± 71.4 in moderate, 281.82 ± 89.5 in severe, and 350.00 ± 95.4 in very severe cases (F = 27.83; p < 0.001). The duration of smoking also showed a consistent rise from 11 ± 4.8 years in mild to 24 ± 7.2, 29 ± 8.3, and 30 ± 9.1 years in moderate, severe, and very severe categories (F = 22.47; p < 0.001). Regarding comorbidities, type II diabetes mellitus was present in six (30%), 15 (38.5%), 10 (45.5%), and four (57.1%) patients across increasing severity stages (χ² = 3.72; p = 0.29), while systemic hypertension was reported in 11 (55%), 23 (59%), 16 (72.7%), and six (85.7%) patients, respectively (χ² = 5.64; p = 0.13). Though not statistically significant, both comorbidities demonstrated an increasing trend with COPD severity (Table 4).

**Table 4:** Smoking and comorbid profile among COPD stages Chi-squared/ANOVA test *A p-value of <0.05 is statistically significant ANOVA: analysis of variance; COPD: chronic obstructive pulmonary disease

Parameter	Mild (n = 20)	Moderate (n = 39)	Severe (n = 22)	Very severe (n = 7)	Test statistic	P-value
Current/former smokers n (%)	13 (65%)	30 (76.9%)	19 (86.4%)	7 (100%)	χ² = 6.84	0.042*
Mean cigarettes per day ± SD	3.8 ± 1.4	6.9 ± 2.1	8.4 ± 2.6	9.1 ± 2.8	F = 14.62	<0.001*
Mean smoking index ± SD	66.75 ± 25.8	213.33 ± 71.4	281.82 ± 89.5	350.00 ± 95.4	F = 27.83	<0.001*
Mean duration of smoking (years) ± SD	11 ± 4.8	24 ± 7.2	29 ± 8.3	30 ± 9.1	F = 22.47	<0.001*
Type II diabetes mellitus n (%)	6 (30%)	15 (38.5%)	10 (45.5%)	4 (57.1%)	χ² = 3.72	0.29
Systemic hypertension n (%)	11 (55%)	23 (59%)	16 (72.7%)	6 (85.7%)	χ² = 5.64	0.13

The mean NT-proBNP levels increased progressively with the severity of airflow limitation among COPD patients, rising from 7.20 ± 1.20 µg/dL in the mild group to 175.71 ± 83.94 µg/dL in the very severe group. This gradation demonstrated a strong positive correlation between NT-proBNP levels and COPD severity. The difference across groups was statistically significant (F = 68.24; p < 0.001), suggesting that elevated NT-proBNP reflects increasing cardiac strain and pulmonary hypertension commonly associated with advanced COPD stages (Table 5).

**Table 5:** NT-proBNP levels across stages of airflow obstruction ANOVA test *A p-value of <0.05 is statistically significant ANOVA: analysis of variance; NT-proBNP: N-terminal pro-B-type natriuretic peptide

Stage of airflow obstruction	N	Mean NT-proBNP (µg/dL)	SD	95% CI (lower-upper)	F-value	P-value
Mild	20	7.20	1.20	6.64-7.76	68.24	<0.001
Moderate	39	11.42	2.06	10.76-12.09		
Severe	22	25.77	9.47	21.57-29.97		
Very severe	7	175.71	83.94	98.09-253.34		

ECG abnormalities demonstrated a progressive increase in prevalence with the advancing severity of airflow obstruction. P pulmonale was absent in the mild group but observed in eight (36.36%) patients with moderate, 10 (48.63%) with severe, and six (66.67%) with very severe COPD. Similarly, RVH was found in nine (39%), 11 (50%), and seven (73.33%) patients across moderate, severe, and very severe groups, respectively. RAD was noted in 10 (45.45%), 12 (56.81%), and five (83.33%) patients in these respective groups. P pulmonale with rightward shift (PPRW) was recorded in two (9.10%) moderate, five (21.18%) severe, and two (33.18%) very severe cases. Low voltage complexes occurred in one (5.70%), four (20.45%), and three (51.61%) patients, while incomplete right bundle branch block (IRBBB) was seen in three (13.63%) severe and two (26.67%) very severe COPD patients (Table 6).

**Table 6:** ECG findings across stages of airflow obstruction RVH: right ventricular hypertrophy; IRBBB: incomplete right bundle branch block; RAD: right axis deviation; PPRW: P pulmonale with rightward shift; ECG: electrocardiogram

Stage of airflow obstruction	P pulmonale n (%)	RVH n (%)	IRBBB n (%)	Low voltage complex n (%)	RAD n (%)	PPRW n (%)
Mild (n = 20)	0 (0%)	0 (0%)	0 (0%)	0 (0%)	0 (0%)	0 (0%)
Moderate (n = 39)	8 (36.36%)	9 (39%)	0 (0%)	1 (5.70%)	10 (45.45%)	2 (9.10%)
Severe (n = 22)	10 (48.63%)	11 (50%)	3 (13.63%)	4 (20.45%)	12 (56.81%)	5 (21.18%)
Very severe (n = 7)	6 (66.67%)	7 (73.33%)	2 (26.67%)	3 (51.61%)	5 (83.33%)	2 (33.18%)

**Figure 1:**
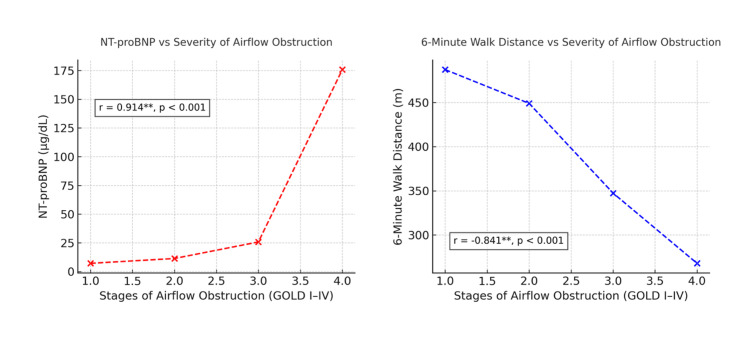
Scatter plot of the relationship between disease severity, NT-proBNP levels, and 6MWD among study patients **A p-value of <0.001 is highly significant 6MWD: six-minute walk distance; NT-proBNP: N-terminal pro-B-type natriuretic peptide; GOLD: Global Initiative for Obstructive Lung Disease

The scatter plot illustrates the relationship between disease severity, NT-proBNP levels, and 6MWD among patients with stable COPD. The left panel shows a strong positive correlation (r = 0.914; p < 0.001) between NT-proBNP levels and the stages of airflow obstruction, indicating that plasma NT-proBNP concentrations rise progressively with increasing COPD severity from GOLD I to GOLD IV. Conversely, the right panel demonstrates a strong negative correlation (r = −0.841; p < 0.001) between 6MWD and airflow obstruction stages, reflecting a marked decline in exercise tolerance as airflow limitation worsens. Together, these findings highlight the complementary nature of NT-proBNP elevation and reduced six-minute walk performance as indicators of physiological and functional impairment in COPD (Figure 1).

## Discussion

COPD is a progressive, debilitating respiratory disorder that primarily affects individuals in later decades of life. In the present study, the mean age of participants was 62.28 years, consistent with prior literature indicating that COPD manifests predominantly in late adulthood due to the cumulative burden of oxidative stress, airway remodeling, and chronic inflammation that accrues over decades of exposure to inhaled toxins.

Our study reaffirmed the pronounced male predominance in COPD, with 80.4% of participants being men, among whom 88% were active or former smokers. This finding mirrors global epidemiologic data that attribute higher COPD prevalence in males to the historically greater prevalence of tobacco use and increased occupational exposures to airborne irritants [15]. Biological sex differences in airway caliber, hormonal influence on lung repair mechanisms, and differential inflammatory responses to smoke further compound this susceptibility. Conversely, among female participants, who constituted a smaller fraction of our cohort, none were smokers; however, 88% reported significant exposure to indoor air pollution due to traditional cooking practices involving wood and cow-dung combustion. This observation underscores a growing body of evidence implicating biomass fuel exposure as a major etiologic factor for COPD in women, particularly in low- and middle-income countries [16].

While smoking is a well-known risk factor for COPD, its correlation with airflow obstruction was evaluated in this study specifically to quantify its impact within our patient population, rather than to restate established knowledge. The significant association observed strengthens the internal validity of our findings by confirming consistent exposure-response patterns. Our study demonstrated a statistically significant positive correlation between smoking indices, including the number of cigarettes smoked per day, the cumulative duration of smoking, and the calculated smoking index, and the severity of airflow obstruction. These findings are concordant with previous population-based studies that have established a dose-response relationship between cumulative tobacco exposure and FEV₁ decline [17]. The results reinforce the critical need for aggressive tobacco-control policies and cessation interventions to mitigate the escalating global burden of COPD.

While outdoor air pollution has traditionally received greater attention, the insidious contribution of indoor air pollution deserves equal scrutiny. Households utilizing biomass fuels for cooking and heating generate high concentrations of particulate matter and carbonaceous gases that can penetrate deep into the lungs, inducing chronic bronchial inflammation and small-airway fibrosis [18]. Our study revealed that a significant proportion of female patients had chronic exposure to biomass fuel combustion, further affirming the need for gender-sensitive preventive strategies and public health policies targeting clean energy adoption.

Comorbid conditions substantially influence the clinical course, outcomes, and management complexity of COPD. The systemic inflammation associated with COPD extends beyond the lungs, contributing to a wide array of extrapulmonary manifestations. In our cohort, cardiovascular comorbidities such as systemic hypertension and ischemic heart disease were highly prevalent, paralleling global evidence of shared risk factors, namely, smoking, sedentary lifestyle, and oxidative stress, and mutual pathophysiologic pathways [19]. Chronic hypoxemia and pulmonary vascular remodeling can exacerbate right-sided cardiac load, predisposing to cor pulmonale and cardiac dysfunction. Likewise, metabolic disorders including diabetes mellitus were common, supporting emerging data that link systemic inflammation and hypoxia-induced insulin resistance to the metabolic complications of COPD [20]. Mental health comorbidities, though not directly measured in our study, have been documented elsewhere to contribute to poor adherence, higher symptom burden, and decreased quality of life [21]. These findings underscore the importance of integrated care models involving pulmonologists, cardiologists, endocrinologists, and mental health specialists.

Symptom burden in COPD is multidimensional, encompassing dyspnea, chronic cough, sputum production, and fatigue. The CAT has become an indispensable tool in quantifying this burden. In our study, mean CAT scores increased progressively with disease severity, from 8.4 in mild airflow obstruction to 27.1 in very severe cases, demonstrating a clear gradient correlating with spirometric decline. These results align with previous reports that link higher CAT scores to reduced health-related quality of life, increased exacerbation risk, and diminished physical performance [22]. The CAT score, therefore, serves not only as a symptom quantifier but also as a surrogate marker for disease progression.

Beyond pulmonary dysfunction, COPD imposes significant extrapulmonary limitations, most notably reduced exercise tolerance. The 6MWT remains a reliable and practical measure of functional capacity and overall cardiopulmonary reserve [12]. Our study revealed a marked decline in 6MWD with advancing airflow obstruction, reinforcing the role of exercise testing in disease staging and prognostication. Compared to the findings of Chen et al. [23], our cohort consistently demonstrated lower mean distances across all GOLD categories, suggesting comparatively poorer functional capacity. The correlation between FEV₁ and 6MWD was strongly negative (r = -0.841; p < 0.001), confirming that greater obstruction translates to reduced exercise tolerance. This observation reflects the cumulative impact of ventilatory limitation, dynamic hyperinflation, and peripheral muscle dysfunction in advanced COPD.

ECG abnormalities provide valuable insights into cardiopulmonary interactions in COPD. In our study, the prevalence and pattern of ECG changes were closely linked with disease severity. RAD, RVH, and P pulmonale were the most frequent findings, reflecting chronic pressure overload of the right ventricle due to pulmonary hypertension [24]. Patients with severe and very severe airflow limitation exhibited a higher frequency of these changes, aligning with observations by Larssen et al. and Kumar et al. [24,25]. These electrophysiologic alterations, when interpreted alongside echocardiographic and biomarker data, enhance the detection of subclinical cor pulmonale in COPD.

Among circulating biomarkers, NT-proBNP has garnered growing attention for its diagnostic and prognostic value in COPD. This peptide, synthesized by cardiac myocytes in response to increased wall stress, reflects underlying ventricular strain and hemodynamic load [26]. In our study, NT-proBNP levels rose progressively with the severity of airflow obstruction, showing a robust positive correlation (r = 0.914; p < 0.001). The mean NT-proBNP concentration escalated from 7.2 µg/dL in mild COPD to 175.7 µg/dL in very severe stages, underscoring its potential as a biochemical indicator of cardiopulmonary compromise. These findings are consistent with those of Mansour et al. [14], who demonstrated that plasma BNP levels correlate significantly with disease severity and predict chronic respiratory failure progression in stable COPD. Similarly, Tyagi et al. reported that elevated NT-proBNP levels predicted longer intensive care stays and greater need for invasive ventilation during acute exacerbations [27]. Nicolae and Ecaterina further reinforced the role of natriuretic peptides as markers of cardiopulmonary remodeling in elderly COPD patients, emphasizing their utility in identifying right heart strain even in the absence of overt cardiac disease [28]. Collectively, these studies, together with our findings, suggest that NT-proBNP measurement may augment clinical evaluation by reflecting the intersection of cardiac and pulmonary dysfunction in COPD.

The present study has several limitations. First, it was conducted at a single tertiary care center with a relatively small sample size, which may limit the generalizability of the findings. Second, the cross-sectional design precludes the assessment of temporal relationships or causal inference between NT-proBNP elevation and disease progression. Third, echocardiographic confirmation of cardiac dysfunction was not systematically performed, which may have provided further insights into right ventricular strain and pulmonary hypertension. Additionally, potential confounders such as renal function and subclinical ischemic heart disease, both of which can influence NT-proBNP levels, were not fully controlled. Future longitudinal, multicenter studies integrating imaging, biomarkers, and serial lung function testing are warranted to validate and expand upon these findings.

Despite these limitations, the study contributes valuable insights into the multifactorial nature of COPD. The observed correlations between NT-proBNP levels, airflow obstruction, and exercise tolerance highlight the interdependence of pulmonary and cardiac function. Incorporating NT-proBNP measurement into routine COPD assessment could aid in the early identification of cardiopulmonary compromise, guide therapeutic optimization, and improve risk stratification. Furthermore, the demonstration of significant ECG alterations across disease stages supports the integration of non-invasive cardiac monitoring into COPD management algorithms. Public health implications extend to smoking cessation advocacy and the mitigation of indoor air pollution, particularly among women in low-resource settings. In conclusion, NT-proBNP serves as a promising biomarker for assessing disease severity and cardiac involvement in stable COPD, warranting further exploration as part of a multidimensional assessment strategy that encompasses spirometry, exercise testing, and cardiovascular evaluation.

## Conclusions

The present study underscores the intricate interplay between pulmonary and cardiovascular dysfunction in stable COPD. Advancing disease severity was associated with the progressive elevation of NT-proBNP levels, increased prevalence of ECG abnormalities, and a significant decline in exercise tolerance, reflecting the cumulative cardiopulmonary burden. The strong positive correlation between NT-proBNP levels and the degree of airflow obstruction, together with the negative association with 6MWD, highlights the biomarker's potential role as a non-invasive indicator of both disease severity and underlying cardiac strain. Incorporating NT-proBNP assessment alongside routine spirometry and ECG may enhance risk stratification, facilitate the early detection of cardiac involvement, and promote more individualized management strategies for COPD patients.
